# Body mass index influences infliximab post-infusion levels and correlates with prospective loss of response to the drug in a cohort of inflammatory bowel disease patients under maintenance therapy with Infliximab

**DOI:** 10.1371/journal.pone.0186575

**Published:** 2017-10-26

**Authors:** Franco Scaldaferri, Daria D‘Ambrosio, Grainne Holleran, Andrea Poscia, Valentina Petito, Loris Lopetuso, Cristina Graziani, Lucrezia Laterza, Maria Teresa Pistone, Silvia Pecere, Diego Currò, Eleonora Gaetani, Alessandro Armuzzi, Alfredo Papa, Giovanni Cammarota, Antonio Gasbarrini

**Affiliations:** Fondazione Policlinico A. Gemelli—Univesità Cattolica del Sacro Cuore, Rome, Italy; Case Western Reserve University, UNITED STATES

## Abstract

**Introduction:**

Infliximab is an effective treatment for inflammatory bowel disease (IBD). Studies differ regarding the influence of body mass index (BMI) on the response to infliximab, with the majority of studies indicating that increased BMI may be associated with a poorer response to Infliximab. However, the pharmacokinetic mechanisms causing this have not yet been reported.

**Aims:**

Examine the correlation between BMI/immunosuppressant use with clinical response, trough and post-infusion levels of infliximab, tumour necrosis factor-α(TNF-α) and anti-drug antibodies(ATI), and determine if these factors can predict future response.

**Methods:**

We collected serum from 24 patients receiving Infliximab before and 30 minutes following infusion. Clinical parameters were collected retrospectively and prospectively. ELISA measurements of infliximab, TNF-α and ATI were performed.

**Results:**

We confirmed that patients with higher infliximab trough levels have a better response rate and that patients with an elevated BMI display a higher rate of loss of response (20%). Patients with a higher BMI had elevated post-infusion levels of infliximab. Additionally, the ratio of IFX/TNF-α trough levels correlated with clinical response to the following infusion.

**Conclusion:**

This study confirms that an elevated BMI is associated with a poorer response to infliximab. For the first time, we describe that a higher BMI correlates with higher post-infusion levels, however this does not correlate with a higher rate of response to the drug, suggesting that circulating drug levels do not correlate with tissue levels. Furthermore, in our small cohort of patients, we identified a possible predictive marker of future response to treatment which may be used to guide dose escalation and predict non-response to infliximab.

## Introduction

Inflammatory Bowel Disease (IBD); Ulcerative Colitis (UC) and Crohn’s disease (CD), occurs due to a complex interaction between the immune system, microbiome, and several environmental factors, in a genetically predisposed individual. The major focus of treatment development has been in targeting the immune response, particularly Tumour Necrosis factor (TNF) -α. Over the last decade, Infliximab (IFX) an anti-TNF-α drug, has dramatically altered the natural history of IBD, delaying the need for surgery, improving quality of life, and reducing inpatient admissions for IBD [1,2]. However, not all patients initially respond to IFX, and a much larger percentage, up to 40%, develop loss of response (LOR) within a year of initiation [3]. This is thought to be due to factors lowering circulating levels of the drug, increasing drug clearance, and the development of anti-Infliximab antibodies (ATIs). LOR requires either dose escalation, a decrease in dosing interval, or the addition of an immunosuppressant. However, these measures all increase the risk of potentially serious side effects. Much work has been done to determine how immunogenicity, the development of anti-drug antibodies, and LOR occurs, to predict response to therapy at an earlier course of the disease [4–6]. It has been shown that IFX trough levels, rather than the absolute presence or level of ATIs seems to be most reflective of response. Several factors are thought to influence trough levels and the production of ATIS, with immunosuppressants proven to increase trough levels and reduce the formation of ATIs [7].

Obesity is recognised as a chronic low grade inflammatory condition which is increasing worldwide [8]. According to the World Health Organisation, up to 70% of European Union residents are overweight, with approximately 30% obese [9]. In parallel to this, there has been an increase in the rate of obesity in IBD patients with up to 50% having a Body Mass Index (BMI) within the obese range (BMI >30) [10,11]. An increased BMI has been shown to be a risk factor for a worse prognosis in IBD, with equivocal reports on the impact of obesity on the response to various medications [12–14]. There is no consensus on the influence of obesity on the response to IFX in IBD, however several studies in Rheumatic conditions have shown that it is associated with an earlier LOR, decreased trough levels, and an improvement in clinical efficacy of IFX following weight loss in obese patients [15–19]. However, these studies have been mainly based on retrospective studies and have evaluated trough levels only.

The pharmacokinetics of infliximab, as of for all drugs administered intravenously, does not depend only on trough levels but also on post-infusion levels, distribution volume and clearance mechanisms, including dose of the target molecule and auto-antibodies. These parameters may all have a potential role, particularly in patients with a higher BMI.

## Aims

1. To examine the correlation between BMI, body fat and the use of immunosuppressants, with serum concentrations of IFX, TNF-α and ATI before and after IFX infusion by measuring trough and post-infusion levels.
2. To investigate the correlation between serum trough and post-infusion levels of IFX, TNF-α and ATIs, with the clinical response retrospectively and prospectively.

## Methods

A total of 24 patients with a diagnosis of CD or UC currently being treated with IFX, attending the Policlinico Agostino Gemelli, were recruited to the study. Only patients receiving maintenance treatment of Infliximab at a stable dose for at least 14 weeks were eligible for inclusion. Ethical approval for the study was granted by the ethics committee of the Fondazione Policlinico Gemelli, and patients aged 18 years and over were recruited to the study after providing written informed consent. IFX treatment was prescribed as per the ECCO guidelines [20,21].

The following definitions were used to describe points of assessment: T0: patients attended to receive an infusion of Infliximab at which point serum was collected, Tpre: the infusion prior to T0, and Tpost: the infusion following T0. LOR pre: LOR between Tpre and T0. LOR post: LOR between T0 and Tpost.

### Clinical assessment

Patients were evaluated clinically at T0, Tpre and Tpost, with the clinical characteristics of Tpre collected retrospectively and those at T0 and Tpost collected prospectively. Data was collected regarding demographics, Montreal classification, medication usage, disease activity, dose of IFX at T0, Tpre and Tpost and dosing interval, BMI and total body fat quantity measured by DEXA at T0. Patients were classified into two groups; monotherapy and concomitant immunosuppressive therapy at T0, Tpre and Tpost. Activity of disease was calculated using the Harvey Bradshaw Index (HBI) for CD and the disease activity index (MAYO) score for UC at T0, Tpre and Tpost. In those patients with a LOR (defined as an increase in the HBI or MAYO score of >2 points) between Tpre and T0 and between T0 and Tpost either: the interval between infusions was reduced, an immunosuppressive therapy was added, or the IFX dose was doubled from 5mg/kg to 10mg/kg.

### Serological assessment

Serum and EDTA samples were collected at two intervals, both at the T0 infusion (prior to infusion–trough levels, and 30mins following infusion–post-infusion levels). Commercially available ELISA kits (Immundiagnostik, Germany) were used to measure the following factors: IFX trough and post-infusion levels, TNF-α trough and post-infusion levels, and ATI trough and post-infusion levels. Following this we calculated the Delta IFX = (IFX post-infusion level—IFX trough level), Delta TNF-α = (TNF-α post-infusion level—TNF-α trough level), Delta ATI = (ATI post-infusion level -ATI trough level), Ratio Pre = (IFX trough level)/(TNF-α trough level), and Ratio post = (IFX post-infusion level)/(TNF-α post-infusion level).

### Statistical analysis

Data was analysed using the statistical software IC STATA12 for the MAC. Distribution of data was compared using the Shapiro-Wilk test and inferential analysis was performed using non-parametric tests for continuous variables; the Spearman test of correlation, Mann-Whitney test and logistic regression models. The null hypothesis was rejected for a p value <0.05.

## Results

### Patient demographics and disease characterisation

Of the 24 patients in our study 19 had CD and 5 had UC. Patient clinical factors are outlined in Table 1. The mean BMI overall was 25.9kg/m^2^ (20.3–47.1), with 50% (n = 12) in the overweight category (BMI>25), and 21% (n = 5) obese (BMI>30), and the mean total body fat was 23.4kg. During the study period 4 (17%) patients had a LOR to treatment between Tpre and T0.

**Table 1:** Disease characterisation, medications and patient demographics.

		Total patients n = 24	Crohn’s disease n = 19	Ulcerative colitis n = 5
**Gender**	Male	18	15	3
	Female			
**Age**	>40 years	12	9	3
	<40 years	12	10	2
**BMI**	>25	12	9	3
	<25	12	10	2
**Immunosuppressant**	Yes	4	4	0
	No	20	15	5
**Dose**	5mg/kg	21	16	5
	10mg/kg	3	3	0
**Interval**	8 weeks	16	15	1
	6 weeks	4	1	3
	4 weeks	4	3	1
**Loss of response**	At infusion	4	4	0
	Post infusion	3	2	1
**Disease activity**	Pre infusion	4	3	1
	At infusion	5	4	1
	Post infusion	4	3	1
**Surgery**	Yes	7	7	0
	No	17	12	5
**Disease localization**			Ileal = 8 colonic = 3 ileocolonic = 8 perianal = 4	Proctitis = 0 left-sided = 5 pancolitis = 0
**Disease phenotype**			Inflammatory = 5 fistulating = 5 stenosing = 9	

### The role of total body fat and BMI

As shown in Table 2, the role of BMI on the levels of each of the parameters measured by ELISA was assessed. Using Spearman’s correlation coefficient, we found that a higher BMI correlated with higher post-infusion IFX levels (ρ = 0.5345 p<0.05) and Delta IFX (ρ = 0.5493 p<0.05), with no significant correlation between BMI and IFX trough levels (ρ = 0.1780). In addition, we found a trend towards a correlation between a higher BMI and higher ATI trough levels, but this was not statistically significant. We found similar results when evaluating the role of body fat, where there was a trend towards higher levels of post-infusion IFX, Delta IFX, and ATI trough levels. However, none of these levels were statistically significant.

**Table 2:** Serum levels of each of the measured factors in the group overall and according to BMI. P value represents significance of comparison of levels based on BMI.

	IFX trough ug/mL	IFX post-infusion ug/mL	Delta IFX ug/mL	TNF trough pg/mL	TNF post-infusion pg/mL	ATI trough AU/mL	ATI post-infusion AU/mL
Total group n = 24	7.0	162.5	155.5	80.5	57.6	48.9	15.9
BMI>25 n = 12	8.98	193	184.4	91.1	60.4	61.4	22.4
BMI<25 n = 12	5.6	140	134.8	74.9	56.0	39.9	11.2
P value	0.37	<0.04*	<0.003*	0.70	0.87	0.52	0.45

* means value is statistically significant.

We evaluated the influence of BMI and body fat on: pre-response and post-response, which showed that BMI and body fat do not have an influence on the pre-response (p = 0.07 and p = 0.15 respectively), but do influence the post-response (z = 0.5408, p<0.04, 95% Confidence Interval (CI) 23.5–28.3, and z = 0.6945 p<0.03, 95% CI 19406–27477, respectively). In those with an elevated BMI and body fat there was a higher probability of LOR to the drug in the following infusion. Using logistic regression analysis, we found that that at equal IFX trough levels, patients with a higher BMI and body fat had an increased likelihood of LOR post-infusion of 20% (p = 0.04, 95% CI 1.4–237738.6).

### The role of immunosuppressants

As shown in Table 3 we evaluated the influence of the use of immunosuppressants on concentrations of each of the measured parameters. This showed that the use of an immunosuppressant is associated with higher IFX post-infusion levels (p<0.03, 95% CI 142.8–182.2) and Delta IFX levels (p<0.01, 95% CI 137.6–173.4). We also showed that the use of an immunosuppressant is associated with lower values of ATI trough and post-infusion levels, however this data was not statistically significant.

**Table 3:** Serum levels of each factor based on use of immunosuppressant.

	IFX trough ug/mL	IFX post-infusion ug/mL	Delta IFX ug/mL	TNF trough pg/mL	TNF post-infusion pg/mL	ATI trough AU/mL	ATI post-infusion AU/mL
Yes	4.5	208.3	203.8	81.8	60.5	29.7	6.4
No	7.5	153.4	145.8	80.4	57.2	52.7	17.8
P value	0.53	<0.03*	<0.01*	0.98	0.94	0.60	0.56

* means value is statistically significant.

### The role of circulating IFX, TNF-α and ATI levels on each other and disease activity

We firstly assessed the influence that ATI trough and post-infusion levels had on IFX trough and post-infusion levels. Using ATI as a dichotomous variable, with a level of <10AU/ml as a cut-off for a negative value, we found that in patients with negative ATI trough levels, the mean values of IFX trough levels and IFX post-infusion levels were significantly higher than in patients with positive ATI trough levels at (12.72ug/mL v 3.57ug/mL, p = 0.01) and (187.7ug/mL vs 147.4ug/mL, p<0.02) respectively.

Next, we evaluated the role of concentrations of each of the parameters on the disease activity at Tpre, T0 and Tpost. We found no correlation between activity of disease at T0, Tpre or Tpost, and any of the concentrations of IFX, TNF-α or ATI, as shown in Table 4.

**Table 4:** Serum levels of each factor based on disease activity at each interval and according to pre-or post-response to IFX.

		IFX trough ug/mL	IFX post infusion ug/mL	TNF trough pg/mL	TNF post-infusion pg/mL	ATI trough AU/mL	ATI post infusion AU/mL
Disease activity pre infusion	Active	6.8	158.2	92.0	54.2	45.2	11.6
	Inactive	7.5	175.5	53.8	59.3	60.1	28.7
Disease activity at infusion	Active	9.0	171	89.1	68.1	31.0	7.0
	Inactive	2.1	142	64.7	37.9	91.2	37.5
Disease activity post infusion	Active	7.9	156.8	96.2	63.1	44.9	12.0
	Inactive	5.3	173.9	57.1	49.3	56.9	23.6
Drug response pre infusion	Yes	7.9	164.9	83.2	36.9	43.6	14.3
	No	2.6	150.8	65.4	61.2	75.4	23.7
Drug response post infusion	Yes	7.5	162.8	74.3	53.7	52.8	17.5
	No	3.6	160.7	115.9	79.3	21.8	4.8

In addition, for the group overall, we found a trend towards a correlation between increased TNF-α trough levels and ATI trough levels with an increased risk of a LOR between Tpre and T0. In contrast, increased in IFX trough levels reduced this risk. However, these results were not found to be statistically significant. In terms of LOR post, we found a trend towards an increased risk with increased TNF-α trough and post-infusion levels, and a reduced risk of LOR post with increased post infusion IFX levels. However, again these results were not found to be statistically significant.

### The role of the ratio of IFX trough levels/TNF-α trough levels pre, and post infusion

**Fig 1:**
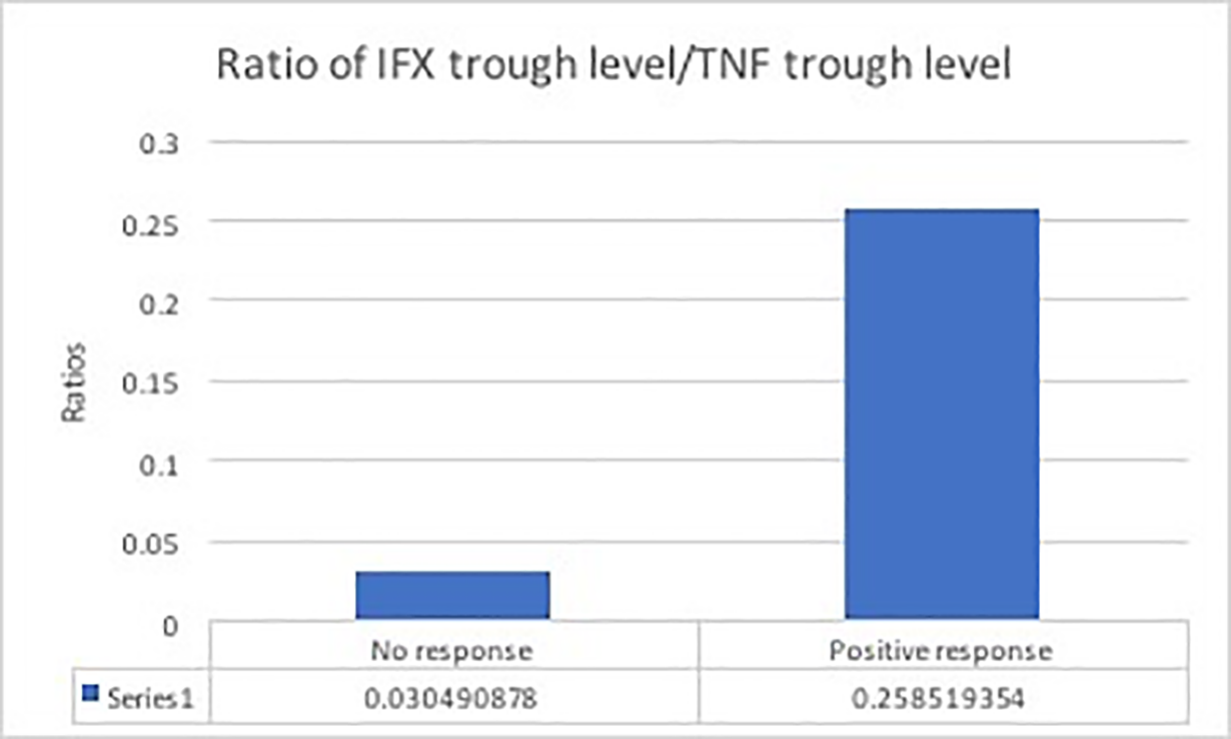
The ratio of trough levels of IFX/TNF-α on the drug response to the following infusion.

We assessed the influence of values of the ratio of IFX/TNF-α pre-and post-infusion on the post-response. The value of the ratio post infusion did not seem to influence the post-response, however, we found that the value of the ratio pre, did influence on the post-response (z = 2.44, p<0.015), as shown in Fig 1. Patients with higher values of the ratio pre-infusion had a lower probability of LOR to IFX between T0 and Tpost.

## Discussion

Biologic treatments, particularly anti-TNF-α agents are the most effective medications for moderate-severe forms of IBD, however despite their efficacy they have a considerable risk of serious adverse events, are expensive, and have a relatively high rate of non-response and LOR, particularly in certain patient groups. The identification of both clinical factors and certain biomarkers which are predictive of response would be of great advantage in directing treatment changes at an earlier stage to expedite clinical improvement for patients and reduce the risk of adverse events, and from an economic perspective for the health system.

There have been several studies which have identified elevated BMI and body fat as risk factors for both disease severity and a poorer response to anti-TNF-α treatment, however, the pharmacokinetic mechanisms by which this occurs remain unclear [12,15,22, 25]. This study has confirmed this association by showing a high rate of LOR, of 20% in patients, with a higher BMI and body fat level, and is the is the first to measure the role of post-infusion levels of drug, cytokine or antibodies rather than solely depending on trough levels. We found that in patients with an increased BMI, the post-infusion values of Infliximab are higher than those with a normal BMI; which suggests that the drug remains centrally in the systemic circulation at higher levels and is less likely to be distributed in tissue. As a result, you would expect to find a better clinical response, however this is not the case. This can be explained by considering the pharmacological structure of IFX; due to its hydrophilic and polar nature, little of its deposition occurs in adipose tissue. The post-infusion levels of the medication increase due to the increased weight-based dose administered, leading to a major central volume of distribution. However, when trough levels are measured, there is no longer an increase in the elevated BMI group. This may be explained by the fact that in the weeks following an infusion other intervening factors for example the formation of ATIs or other antibodies which also increase with BMI, lead to a decrease in circulating IFX levels. In addition, there remains the possibility of activation of alternative inflammatory pathways which are independent of TNF-α, which has been demonstrated by other studies, as it has been shown that TNF-α is not as dramatically elevated in obese IBD patients [23]. These finding are interesting due to the recommended weight-based dosing regimen of IFX, which if not warranted may cause more harm than benefit, particularly due to the already impaired immune system of obese patients. Further delineating predictive factors for primary non-responders and LOR to in patients with an elevated BMI is of great importance, to prevent increasing the central drug volume and the associated risks of this unnecessarily.

In addition, we have shown that the use of immunosuppressants is associated with higher post-infusion levels of IFX. This was also associated with, and perhaps explained by the reduced formation of ATIs, which increase the clearance of the drug and activate other pathways of inflammation. Higher post-infusion IFX levels could be a result of reduced clearance of the drug, caused by a reduced formation of ATIs due to the additional immunosuppressant effect. In addition, Azathioprine is known to cause a reduction in other signalling factors which consume anti-TNF-α, for example other antibodies (anti-nuclear antibodies (ANA) and anti-double stranded DNA (dsDNA), along with other pro-inflammatory cytokines [24, 25]. The probability of LOR to the following infusion was shown to be reduced by an increase in post-infusion IFX levels. The concentration of IFX was therefore likely to have been adequate to neutralise the TNF-α from the beginning, immediately following the infusion. This data is of great value as it considers the drug response to the next infusion evaluated in a prospective manner, which is rarely studied in the literature. An increase in values of TNF-α and of ATI trough levels instead, increase the probability of LOR to the following dose of IFX as there is an excess of TNF-α which is not neutralised by the drug, leading to the formation of ATI and ultimately increasing the clearance of the drug.

A further clinically relevant assessment of this study was the evaluation of the use of a ratio of IFX/TNF-α serum levels as biochemical predictive factors for future response to treatment. In patients with higher pre-infusion ratios of IFX trough levels/TNF-α trough levels, there is less of a risk of LOR to the following infusion. This parameter is of great importance as it describes the measurement of IFX in proportion to TNF-α before the evaluation of the clinical response to the drug. It is the first in the literature to try and predict response based on the biology of the drug. This parameter had been demonstrated via a prospective evaluation of clinical response to the next infusion of the drug, a predictor of response to therapy with IFX and therefore can also be considered as a predictor of patients who may become non-responders and in fact, may also be useful in identifying primary non-responders.

Despite the innovative findings in this study, it does however have some limitations. Firstly, the study population is not homogenous, and the number of enrolled patients with a LOR to IFX is low (of 24 patients, only 5 lost response, giving a ratio of 1:5). Another limitation of our study is that the trough and post-infusion levels of IFX, ATI and TNF-α were measured only at T0. To fully understand the pharmacokinetic properties of a drug and to fully understand the role of these factors in clinical practice, it would be better to track the progress of these markers over time. Furthermore, we have not measured markers of other inflammatory pathways aside from TNF-α, which admittedly may not be the sole driver of inflammation in patients with an elevated BMI. Finally, the interval between infusions for patients may have varied based on clinical need, which may have affected levels of measured factors, although they had been stable for at least 2 infusions or 3–4 months prior to inclusion in the study.

## Conclusions

This study has firstly confirmed the previous data in the literature, that patients who have a better response to IFX are those with elevated concentrations of the drug at trough levels, in whom IFX remains at an adequate level to neutralise TNF-α and not activate mechanisms to increase drug clearance. Furthermore, we have confirmed previous suggestions that patients with higher levels of BMI and body fat, have a poorer response to IFX as evidenced by a higher rate of loss of response. Higher BMI seems to particularly influence IFX post-infusion levels, although higher circulating levels do not seem to correlate with higher tissue levels, suggesting that in obese people the drug may have a more rapid clearance.

Finally, for the first time in the literature, we have shown a relationship between a ratio of serum IFX/TNF-α trough levels which may be a useful predictive factor for response to future infusions and identifying primary non-responders. The prognostic role of this relationship needs to be studied further in trials but could represent a crucial parameter in personalising treatment by identifying a specific pathogenic pathway for each patient.

## Supporting information

S1 File (XLSX)
